# A multidimensional physical scale is a useful screening test for mild depression associated with childcare in Japanese child-rearing women

**DOI:** 10.3389/fpsyt.2022.969833

**Published:** 2022-12-01

**Authors:** Mariko Takeuchi, Michiko Matsunaga, Ryuichiro Egashira, Akimitsu Miyake, Fumihiko Yasuno, Mai Nakano, Misaki Moriguchi, Satoko Tonari, Sayaka Hotta, Haruka Hayashi, Hitomi Saito, Masako Myowa, Keisuke Hagihara

**Affiliations:** ^1^Department of Advanced Hybrid Medicine, Osaka University Graduate School of Medicine, Osaka, Japan; ^2^Graduate School of Education, Kyoto University, Kyoto, Japan; ^3^Japan Society for the Promotion of Science, Tokyo, Japan; ^4^Department of Medical Innovation, Osaka University Hospital, Osaka, Japan; ^5^Department of Psychiatry, National Center for Geriatrics and Gerontology, Aichi, Japan

**Keywords:** maternal depression, physical symptoms, postpartum, the multidimensional physical scale (MDPS), the Beck Depression Inventory–Second Edition (BDI-II), Kampo medicine, screening for depression

## Abstract

**Introduction:**

Maternal depression is one of the important problems of postpartum women. For its early detection and appropriate treatment, it is necessary to identify women at high risk for depression quickly and easily.

**Materials and methods:**

A simple screening scale for depression from physical aspects, the multidimensional physical scale (MDPS), which is a 17-item, self-report, three-step scale (0, 1, 2) according to the theory of Kampo medicine, was developed. The aim of the present study was to develop (*n* = 785) and validate (*n* = 350) the MDPS that was designed to rate the risk of depression. The Beck Depression Inventory–Second Edition was used for determination of depression. In the development cohort, the final model was determined using multi-regression logistic analysis.

**Results:**

The components of the MDPS for mothers (MDPS-M) were developed, containing the total score of MDPS (0–34 points) and resumption of menstruation or not (–3, 0 points). Receiver-operating characteristic curve analysis of the MDPS-M (–3 to 34) for identifying a high risk of depression showed moderately good discrimination [area under the curve (AUC) = 0.74, 95% confidence interval (CI): 0.70–0.78]. At the cutoff value of MDPS-M (9/10), its sensitivity, specificity, positive predictive value, and negative predictive value were 84.9, 45.7, 36.7, and 89.2%, respectively. External validation of the MDPS-M showed moderately good discrimination (AUC = 0.74, 95% CI: 0.68–0.79) using the same analysis as the development cohort.

**Conclusion:**

These results indicate that the MDPS-M is a useful, simple, clinical scale for early identification of mothers at high risk of depression in primary care.

## Introduction

Japan has faced a severely declining birth rate and rapidly increasing elderly population (1). The solution for the declining birthrate has been a long-standing issue, but it is currently getting worse. Although various problems are related to the declining birth rate, female psychological stress is one of the most common reasons (2). Around 60% of mothers experience psychological distress (3). Recently, some studies reported that emotional support for mothers was associated with increased fertility (4). In the perinatal period, women’s physical and mental condition changes dramatically due to childbirth, changes in hormonal balance, and so on. It is said that a woman’s body recovers to the prenatal condition from 6 to 8 weeks to more than 6 months after childbirth (5, 6), but many mothers return to work early in conjunction with parenting in Japan (7). In addition, social support for childcare tends to be insufficient (8). As a result, maternal depression increases and has become a social problem (9).

It has been reported that postpartum depression is seen in 10–15% of mothers (10, 11), and, in particular, it was increased to 20–30% during the COVID-19 pandemic (12, 13). A meta-analysis of postpartum depression in Japan reported that the risk ratio of postpartum depression in primiparas is 1.76 (8). Previously, it was thought that the onset of postpartum depression was often several months after childbirth (14). However, recent reports showed that the number of women with depressive symptoms 1 year after childbirth was the almost the same as that in the first month after childbirth. It is especially noteworthy that half of the women with depressive symptoms 1 year after childbirth did not have depressive symptoms 1 month after childbirth (15). There is concern that the depressive symptoms of mothers can be prolonged (16). We considered “depression” as one of the problems to be solved not only in the immediate postpartum period, but also in women of child-rearing age that continues afterward.

Given the above, it is necessary to develop a scale that can quickly identify high-risk mothers who are prone to depressive symptoms even for mothers who were healthy before pregnancy. However, it is difficult to screen for depressive symptoms in mothers, because mothers avoid revealing mental symptoms [National Collaborating Centre for Mental Health (UK), (17, 18)]. About 30% of women with physical or mental distress underwent a medical examination (19). Moreover, only 10% of postnatal mothers with depressive symptoms consulted a specialized agency (20). The Edinburgh Postnatal Depression Scale [EPDS; (21)] was commonly used for screening of postpartum depression. In Japan, its validity has been established for the first month postpartum and its use during pregnancy is also common. However, the use of the EPDS for screening is less common after the second year of postpartum. In addition, several observations reported that the validation results were not stabilized depending on each research environment (22).

In general, doctors engaged in maternal and child medical examinations focus on the health of the children rather than of the mothers in the perinatal period (23). In addition, accurate identification of mothers with depressive symptoms during a time-limited visit may be difficult (24). The conventional mental questionnaire is not always appropriate to judge the mental state of mothers with depressive symptoms. On the other hand, somatic symptoms of depression are seen in primary care worldwide, but it is well known that the frequency of complaints depends on the culture and healthcare system of the country (25). Another aspect is needed to develop a scale that can quickly identify high-risk subjects based on physical symptoms.

Traditional Japanese herbal medicine (Kampo medicine) has been widely applied in the treatment of women in the perinatal period (26–28). In Kampo medicine, various symptoms are clustered according to the original concepts of Kampo medicine. Each symptom does not directly lead to a diagnosis, but by their accumulation, problematic points emerge and are diagnosed from the Kampo medicine point of view. For example, the physician evaluates physical activity, somatic disorders, hormonal activity, microvascular disorders, and meteoropathy-related symptoms. The Kampo formula is subsequently determined according to the Kampo medicine diagnosis.

In a large-scale survey of mothers and children, we developed the multidimensional physical scale (MDPS) as a scale to judge the physical aspects of mothers from the Kampo medicine perspective. The MDPS consists of 17 simple questions and is divided into five clusters: physical activity index, somatic disorder index, hormonal activity index, microvascular disorders index, and meteoropathy-related index. At the same time, the relationship between the MDPS and the Beck Depression Inventory–Second Edition (BDI-II) (29) was investigated. We chose the BDI-II as an index of the state of depression because we investigated the depression of women not only in the first year of postpartum but in the second and more year as a child-rearing age. BDI-II is used in wide range age, not limited to a specific period as the EPDS. Furthermore, the correlation between BDI-II and EPDS is high (30).

In the present large-scale survey of mothers, about 1,200 subjects were divided into a 7 to 3 ratio; one group was used as a development cohort and the other as a validation cohort for accuracy and calibration. The components of the MDPS for mothers (MDPS-M) were developed, containing the total score of MDPS and resumption of menstruation or not. The MDPS-M showed a high correlation with the BDI-II and was found to identify mothers with a high risk of depression from physical aspects. This study clearly demonstrated that the MDPS-M is a useful tool to screen for mothers’ physical and depressive state in the parenting period.

## Materials and methods

### Study design and subjects

The following steps were taken to establish a predictive scale for the early detection of depression. First, through discussions among experts in internal medicine, obstetrics and gynecology, and Kampo medicine, candidate questions for the MDPS were developed (Table 1). Second, a simplified predictive model was generated in the development cohort. Finally, the model was externally validated in a validation cohort. This study followed the TRIPOD (Transparent Reporting of a Multivariable Prediction Model for Individual Prognosis or Diagnosis) reporting guidelines (31).

**TABLE 1:** Candidate questions for MDPS components.

			0: None of the time or rarely	1: Occasionally or sometimes	2: Most or all of the time
PAI	MDPS 1	I get tired more easily than before.	◻	◻	◻
	MDPS 2	I am sleepy after a meal.	◻	◻	◻
	MDPS 3	I feel sluggish in my hands or legs.	◻	◻	◻

SDI	MDPS 4	I have a sensation of a lump or foreign body in the throat.	◻	◻	◻
	MDPS 5	I feel that I haven’t completely emptied my bladder after urination.	◻	◻	◻
	MDPS 6	I feel bloated.	◻	◻	◻

HAI	MDPS 7	My hands and legs feel cold.	◻	◻	◻
	MDPS 8	My hair loss has become serious.	◻	◻	◻
	MDPS 9	I suffer from dry skin.	◻	◻	◻
	MDPS 10	I suffer from vaginal itchiness or dryness.	◻	◻	◻

MDI	MDPS 11	I have bags under my eyes.	◻	◻	◻
	MDPS 12	I have age spots.	◻	◻	◻
	MDPS 13	I have a rough skin.	◻	◻	◻
	MDPS 14	I have hemorrhoids.	◻	◻	◻

MRI	MDPS 15	I have headaches.	◻	◻	◻
	MDPS 16	I feel dizzy.	◻	◻	◻
	MDPS 17	I have swelling.	◻	◻	◻

MDPS, multidimensional physical scale; PAI, physical activity index; SDI, somatic disorders index; HAI, hormone activity index; MDI, microvascular disorders index; MRI, meteoropathy related index. The following is a list of the ways you might have felt. Please select the number how often you have felt this way recently.

**FIGURE 1:**
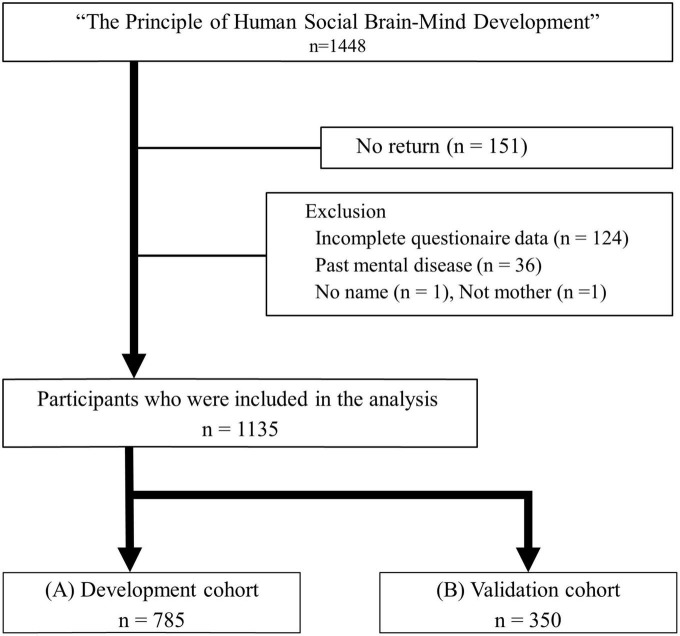
Flowchart of patient enrolment for the development and validation cohorts. **(A)** Development cohort. **(B)** Validation cohort. MDPS, multidimensional physical score.

This study is part of the research project “the Principle of Human Social Brain-Mind Development” in Japan. The data collection was conducted between January and November 2021. To develop and validate the MDPS, 1,448 Japanese postpartum women who were raising children aged 0–5 years in nursery school were recruited throughout Japan. We excluded mothers who are in the hospital due to mental or physical illness or who cannot answer the questionnaire in Japanese. The data of 1,297 of them were finally collected. Of the 1,297 participants’ data, the data of 118 participants were excluded from the analysis for the following reasons: 124 had incomplete answers to the questionnaire, 36 had a history of mental illness, 1 was not the mother (grandmother was the respondents), and 1 had no name. Finally, the data of 1,135 participants were used for the analysis. The holdout technique that randomly selected 70% of the data for the development cohort while holding out 30% of the data for the validation cohort was used. As a result, the data of 785 participants were analyzed for the development cohort and 350 for the validation cohort (Figure 1).

### Depression criteria and related factors of the multidimensional physical scale

Depression was evaluated using the BDI-II, which assesses the severity of depressive symptoms. The BDI-II is based on the diagnostic criteria of the DSM-IV and has been validated and standardized in Japanese (32). It consists of 21 items rated on 4-point scales. To identify individuals with possible depression, the cutoff point was set to 14 points (mild illness) based on the manual. Other information, such as age, previous childbirth, and whether or not menstruation had restarted, was also evaluated using a questionnaire.

### Questions used for the development of the multidimensional physical scale

An expert committee composed of internal medicine, obstetrics and gynecology, and Kampo medicine developed an MDPS scale as a simple, self-report questionnaire based on the theory of Kampo medicine (33). At the same time, problems of postpartum women were selected as candidate questions. Basically, questions that are easy to understand and that can be self-reported by the respondent were selected. Items that could not be evaluated without objective measurement from various perspectives were omitted.

Finally, a consensus was reached to adopt 17 items on five subscales, with each item rated on a 3-point scale (Table 1). The five subscales were (i) physical activity index (PAI) [higher score indicates physical inactivity (e.g., tiredness, physical lethargy)]; (ii) somatic disorders index (SDI) [higher score indicates high physical depression symptoms (e.g., anorexia, bloating)]; (iii) hormone activity index (HAI) [higher score indicates poor hormone function (e.g., coldness, dizziness, dry skin)]; (iv) microvascular disorder index (MDI) [higher score indicates impaired microcirculation related to female hormone function (e.g., skin pigmentation and rough skin)]; and (v) meteoropathy-related index (MRI) [higher score indicates impaired water metabolism and meteorological diseases (e.g., swelling, headache due to bad weather)].

### Statistical analysis

Continuous variables are summarized as means ± standard deviation (SD) or medians (interquartile range), and categorical variables are presented as frequencies and percentages.

To build a prediction model, a multivariate logistic regression model was first fitted with depressive or healthy judged by the BDI-II as the response variable and the total score of the MDPS, age, parity (primiparous/multiparous), and resumption of menstruation (non-resumption/resumption) as explanatory variables. A backward stepwise procedure was then conducted to narrow down the set of predictors. The final model was selected based on the smallest value of the Akaike information criterion (AIC).

To create a simple scale for screening depression, each of the predictors was assigned an integer score proportional to the β coefficients.

The predictive performance of the MDPS for mothers (MDPS-M) was assessed with respect to discrimination and calibration. To quantify discriminatory performance, the receiver-operating characteristic (ROC) curves (34) for area under the curve (AUC) were generated using the development cohort. A clinically relevant cutoff point was determined to be the point at which sensitivity was greater than 80%, and sensitivity, specificity, positive predictive value (PPV), and negative predictive value (NPV) were calculated. Subsequently, calibration was assessed graphically using non-parametric calibration curves with pointwise 95% confidence intervals (CIs) and their graphical summaries (calibration-in-the-large and calibration slope) (35). Flexible calibration curves can be estimated with the locally weighted scatter plot smoother (LOESS) function. If the calibration-in-the-large is 0 and the calibration slope is 1, the predictive model for MDPS-M is perfectly calibrated. The external validity of the MDPS-M was examined using the data of the validation cohort. The ROC curves for AUC were generated in the same way to quantify discriminatory performance. The sensitivity, specificity, PPV, and NPV were calculated using the cutoff determined in the development cohort. Calibration plots were then plotted to compare the predicted probabilities with the actual outcomes.

In each analysis, only cases with no missing values were included. R version 4.1.2 (R Foundation for Statistical Computing, Vienna, Austria) was used for the analyses. ROC curves were plotted with the R package pROC, and calibrations were assessed with the R package rms. A *p*-value of < 0.05 was considered significant. All statistical analyses were performed by independent statisticians.

### Ethical considerations

This study was approved by the Institutional Review Board (IRB) of the Osaka University School of Medicine (20521) and the Kyoto University Graduate School and Faculty of Medicine, Ethics Committee (no. R2624), and was registered in the UMIN system (UMIN000043945). A group briefing session was held for the subjects to explain the outline of the study using prepared materials, and written, informed consent was obtained using a consent form.

## Results

### Subjects’ characteristics

The development cohort included 785 eligible participants, and the validation cohort included 350 eligible participants (Figure 1). In the development cohort, the mean age of the participants was 34.3 ± 4.8 years, and the mean months after birth was 22.8 ± 11.9 months. According to the BDI-II, 212 (27.0%) cases were classified as high-risk subjects: 119 (15.2%) as mild depression, 70 (8.9%) as moderate depression, and 23 (2.9%) as severe depression. In the validation cohort, the mean age of participants was 34.1 ± 4.8 years, and the mean months after birth was 23.6 ± 12.8 months. Overall, 109 (31.2%) were classified as high-risk subjects: 65 (18.6%) as mild, 36 (10.3%) as moderate, and 8 (2.3%) as severe depression. The characteristics of the subjects are shown in Table 2.

**TABLE 2:** Subject characteristics in the development and validation cohort.

	Development cohort (*n* = 785)	Validation cohort (*n* = 350)
Age (years)	34.3 ± 4.8	34.1 ± 4.8
Months after birth (months)	22.8 ± 11.9	23.6 ± 12.8
Height (cm)	158.5 ± 5.7	159.0 ± 5.3
Weight (kg)	54.1 ± 9.7	54.4 ± 9.0
BMI (kg/m^2^)	21.5 ± 3.7	21.5 ± 3.4
Resumption of menstruation, yes (%)	720 (91.7)	323 (92.3)
Parity (proportion of primiparous), *n* (%)	301 (38.3)	128 (37.8)
MDPS total, median (IQR) (0–34)	14 (8)	15 (8)
Physical activity index (PAI) (0–6)	3 (2)	3 (2)
Somatic disorders index (SDI) (0–6)	1 (2)	1 (2)
Hormone activity index (HAI) (0–8)	4 (3)	4 (3)
Microvascular disorders index (MDI) (0–8)	4 (3)	4 (3)
Meteoropathy related index (MRI) (0–6)	3 (2)	3 (2)
BDI-II, median (IQR) (0–63)	9 (9)	10 (10)
Normal (0–14), *n* (%)	573 (73.0)	241 (68.9)
Mild (14–19), *n* (%)	119 (15.2)	65 (18.6)
Moderate (20–28), *n* (%)	70 (8.9)	36 (10.3)
Severe (29–63), *n* (%)	23 (2.9)	8 (2.3)

BMI, body mass index; IQR, interquartile range; MDPS, multidimensional physical scale; BDI-II, beck depression inventory.

### Multivariable logistic analysis of the multidimensional physical scale

**FIGURE 2:**
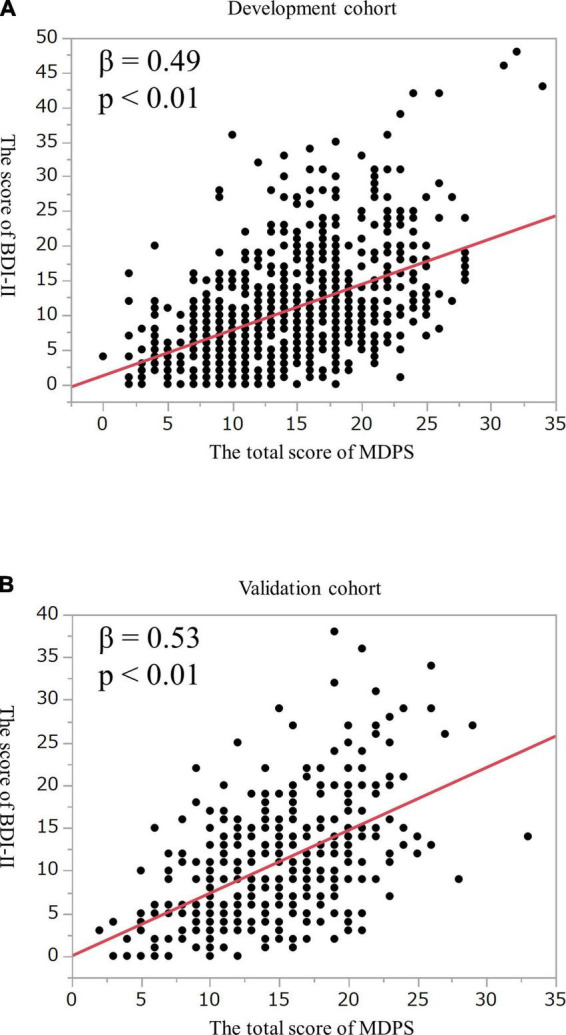
Correlation between the score of the MDPS and the BDI-II in **(A)** the development cohort and **(B)** the validation cohort. MDPS, multidimensional physical score; BDI, beck depression inventory.

The total score of the MDPS showed a positive relationship with the BDI-II score on monovariable regression in both the development and validation cohorts (β = 0.49, 0.53, *p* < 0.01, respectively) (Figure 2).

To create a simplified clinical scale, the relationships between the determination of depression and the score of each of the 17 questions, five subscales, or total of the MDPS were examined by multiple logistic regression analysis in the development cohort. Adjusted odds ratio (ORs) of the 17 questions of the MDPS ranged from 0.85 to 3.22 for mild depression. Though multivariable logistic analysis showed that not all questions of the MDPS were significantly associated with depression based on the BDI-II, the adjusted ORs of the MDPS1 and MDPS3 were 3.22 and 1.57, respectively (*p* < 0.01) (Table 3A). The five subscales were then examined by multiple logistic regression analysis. The adjusted ORs of the five subscales ranged from 1.06 to 1.53 for mild depression. In particular, the adjusted ORs of PAI, SDI, and HAI were 1.53, 1.26 (*p* < 0.01), and 1.13 (*p* = 0.02), respectively, based on the BDI-II (Table 3B). Similarly, the total score of the MDPS was associated with the BDI-II (adjusted OR 1.19, *p* < 0.01). Since the purpose was to develop a simple screening tool for use at the bedside, a suitable model was investigated using the total score of the MDPS and important clinical indicators.

**TABLE 3:** Results of the multivariable regression analysis of the MDPS.

(A)						
		Adjusted OR	95% CI	Coefficient	SE	*P-value*
PAI	MDPS1	3.22	2.21–4.69	1.17	0.19	< 0.01
	MDPS 2	1.02	0.81–1.30	0.02	0.12	0.84
	MDPS 3	1.57	1.23–2.01	0.45	0.13	< 0.01

SDI	MDPS 4	1.30	0.94–1.80	0.26	0.16	0.11
	MDPS 5	1.28	0.91–1.81	0.25	0.17	0.15
	MDPS 6	1.12	0.86–1.48	0.12	0.14	0.40

HAI	MDPS 7	1.13	0.90–1.42	0.12	0.12	0.30
	MDPS 8	1.00	0.81–1.24	0.01	0.11	0.96
	MDPS 9	1.21	0.93–1.57	0.19	0.13	0.15
	MDPS 10	1.36	0.98–1.88	0.31	0.17	0.07

MDI	MDPS 11	1.23	0.98–1.53	0.20	0.11	0.07
	MDPS 12	0.97	0.78–1.21	–0.03	0.11	0.78
	MDPS 13	1.02	0.81–1.28	0.02	0.12	0.89
	MDPS 14	0.97	0.79–1.20	–0.03	0.11	0.81

MRI	MDPS 15	0.85	0.66–1.10	–0.16	0.13	0.22
	MDPS 16	1.24	0.96–1.60	0.22	0.13	0.10
	MDPS 17	1.11	0.88–1.40	0.10	0.12	0.39

**(B)**					

	**Adjusted OR**		**95% CI**	**Coefficient**	**SE**	***P-value***

PAI	1.53		1.34–1.76	0.43	0.07	< 0.01
SDI	1.26		1.10–1.45	0.23	0.07	< 0.01
HAI	1.13		1.02–1.26	0.12	0.05	0.02
MDI	1.06		0.96–1.16	0.06	0.05	0.24
MRI	1.08		0.96–1.22	0.08	0.06	0.22

MDPS, multidimensional physical scale; PAI, physical activity index; SDI, somatic disorders index; HAI, hormone activity index; MDI, microvascular disorders index; MRI, meteoropathy related index; OR, odds ratio; CI, confidence interval; SE, standard error.

### Development of the multidimensional physical scale for mothers

**FIGURE 3:**
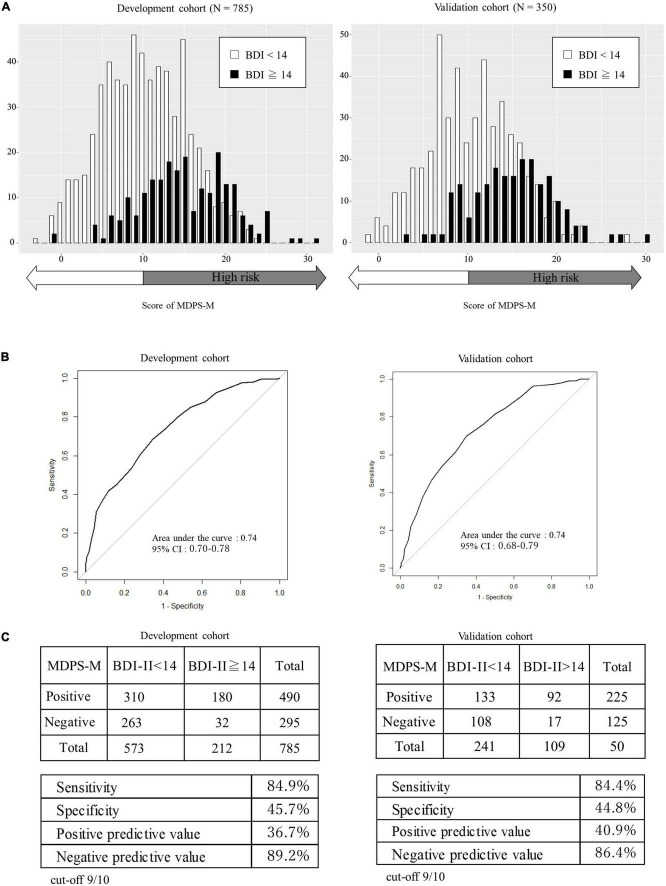
Score distribution, discrimination capability, and diagnostic accuracy of the MDPS-M in the development cohort and the validation cohort. **(A)** Score distribution of the MDPS-M in the development cohort and the validation cohort. **(B)** Discrimination capacity of the MDPS-M for the identification of depression based on the BDI-II by ROC curves. **(C)** Sensitivity, specificity, positive predictive value, and negative predictive value for a score of 10 or more for a high risk for depression. AUC, area under the curve; MDPS-M, multidimensional physical scale for mothers; BDI, beck depression inventory; ROC, receiver-operating characteristic.

**FIGURE 4:**
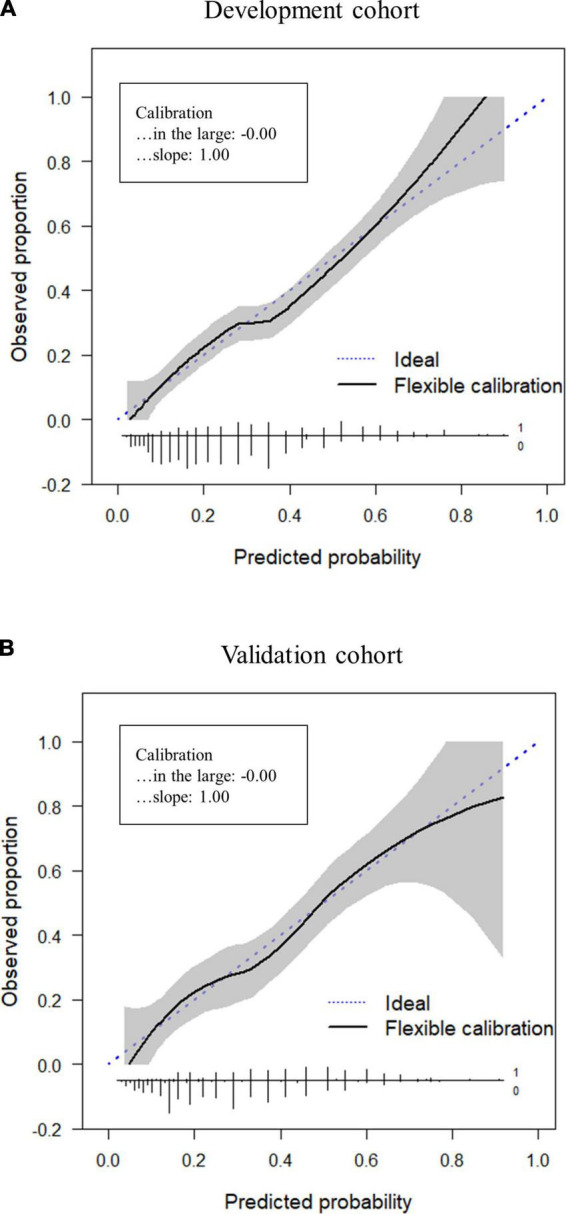
Calibration plots for the development cohort and validation cohort. Calibration is assessed graphically using the non-parametric calibration curves with pointwise 95% confidence intervals, and their graphical summaries (calibration-in-the-large and calibration slope). **(A)** Development cohort. **(B)** Validation cohort.

The total score of the MDPS, age, parity (primiparous or multiparous), and resumption of menstruation (yes or no) were identified as independent variables. A backward stepwise selection method was also performed to reduce the variables, and the total score of the MDPS, age, and resumption of menstruation were identified for the final models. To develop a simple scale, age was excluded because the coefficient was too small. Therefore, the total score of the MDPS and resumption of menstruation were selected as the components of the MDPS-M. Based on the β regression coefficients of the final model, each factor was given an integer weighting: 0–3 points for resumption of menstruation, and 0–34 points for the total MDPS score (Table 4). The MDPS for mothers (MDPS-M) was calculated by a simple sum of these two scores with a minimum of -3 and a maximum of 34 points (Table 4). A higher score indicates a higher risk of depression, and the distribution of scores is shown in Figure 3A. The MDPS-M score distributions were apparently different between mild depression (BDI-II ≥ 14) or not (BDI-II < 14). The ROC curve of the MDPS-M is shown in Figure 3B (AUC = 0.74, 95% CI: 0.70–0.78). Since the clinically relevant cut-off point was defined as the point at which the sensitivity was greater than 80%, the cutoff value of the MDPS-M was determined to be 9/10. MDPS-M’s sensitivity, specificity, PPV, and NPV were then 84.9, 45.7, 36.7, and 89.2%, respectively (Figure 3C). In the calibration plot, the predicted risks showed good agreement with the actual risks of depression with calibration-in-large of 0.00 and calibration slope of 1.00 (Figure 4A).

**TABLE 4:** Final version of the MDPS-M and score calculation method.

Age Parity(0: primiparous, 1: multiparous) Resumption of menstruation(0: without, 1: with) MDPS. total					
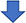	By backward stepwise selection				

**Table:** 

	Adjusted OR	95% CI	Coefficient	SE	Pr
Age	0.97	0.94–1.01	–0.03	0.02	0.11
Resumption of menstruation (yes or no)	0.59	0.33–1.07	–0.52	0.30	0.08
MDPS total	1.19	1.15–1.23	0.17	0.02	<0.01

MDPS, multidimensional physical scale; MDPS-M, MDPS for mothers; OR, odds ratio; CI, confidence interval; SE, standard error; AIC, Akaike information criterion.

**Table:** 

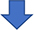	In the final model, ‘age’ was excluded because of very small effect.				
**MDPS-M = MDPS.total-3 × resumption of menstruation (0: without, 1: with)**					

### External validation of the multidimensional physical scale for mothers

To externally validate the MDPS-M derived from the development cohort, discriminant performance and calibration were evaluated in the validation cohort. The score distribution of the validation cohort is shown in Figure 3A, and the ROC curve is shown in Figure 3B (AUC = 0.74, 95% CI: 0.68–0.79). Sensitivity, specificity, PPV, and NPV at the cutoff of 9/10 were calculated to be 84.4, 44.8, 40.9, and 86.4%, respectively (Figure 3C). In the calibration plot, the predicted risk showed moderate agreement with the actual risks of depression, with the calibration-in-large of 0.00 and calibration slope of 1.00 (Figure 4B). The results of external validation of the MDPS-M were almost the same as that of the development of the MDPS-M.

## Discussion

In this study, a simple, self-reported, screening scale for maternal depression from physical aspects was developed according to the theory of “Qi, Blood, and Fluid” of Kampo medicine. The MDPS-M consists of 17 questions and resumption of menstruation. The MDPS-M has a sensitivity of over 80% in detecting a high risk for mild depression in Japanese child-rearing women at the cutoff point of 9/10. This scale is appropriate for use in primary care settings where time can be limited.

Maternal depression is not only a serious social problem, but also a medical problem. Depressive symptoms of mothers are related to child abuse (36–38) and suicide of the mothers themselves (39, 40). Many reports have shown that depressive symptoms in mothers affect the emotions, behaviors, cognitive development, and language development of children (41–44). The quality of a child’s development depends on the mother’s mental state. Thus, maternal depression should be dealt with immediately as an important social and medical problem.

However, it is well known that many patients with depression present with physical symptoms in primary care (45, 46). An online survey of 4,510 adults in the United States found that 60–80% of patients were uncooperative in providing clinicians with important health information (47). In addition, it has been reported that mothers of the parenting generation hesitate to talk about their mental symptoms [National Collaborating Centre for Mental Health (UK), (17, 18)]. In the mother and child examinations, medical staff deal with many subjects, and time for diagnosing depressive symptoms is limited (24). If a scale focusing on physical findings for high-risk patients with depression can be developed for use at the bedside, it is expected to be highly useful. It is well known that the frequency of somatic symptoms depends on the culture and healthcare system of the country (25). Thus, we have to develop a physical scale to take into account the cultural background of Japan.

Kampo medicine is often used for patients in the field of obstetrics and gynecology (26–28). The theory of Qi, Blood, and Fluid is used to evaluate the physical status and to select the Kampo formula against disease (33). Although the theory of Qi, Blood and Fluid has a greater meaning, Qi is a general term for the functions of invisible bioactive substances, cytokines, hormones, neurotransmitters, gastrointestinal peptide hormones, and so on from the modern medicine perspective. The physician evaluates whether the role of invisible bioactive substances is reduced or if they are flowing well. Blood mainly means the functions of sex hormones. Fluid means the distribution and circulation of body fluid volume and reflects the symptoms of so-called meteoropathy. The physician evaluates whether the activity of invisible bioactive substances is reduced or they are flowing well. At the same time, the physician evaluates the activities related to sex hormones and the distribution and circulation of body fluid. Kampo medicine contains unique diagnostic methods, e.g., pulse diagnosis, medical interview, inspection, abdominal examination, and so on, according to the theory of Qi, Blood, and Fluid. In this study, the unique diagnostic methods were excluded, and the MDPS was developed as a simple questionnaire.

The physical activity index (PAI) represents the role of invisible bioactive substances. For example, the PAI reflects the function of ghrelin, which stimulates appetite (48). Rikkunshi-To (RKT) is the one of the representative herbal medicines that improves the role of Qi. RKT is known to induce the active form of ghrelin and improve functional dyspepsia (49, 50). The somatic disorder index (SAI) indicates the flow of invisible bioactive substances. For example, Hange-koboku-To (HKT) increased plasma levels of substance-P, and HKT can prevent aspiration pneumonia in patients after cardiovascular surgery (51, 52). The hormonal activity index (HAI) means the role or amounts of sexual hormones. For instance, Toki-shakuyaku-San (TSS) improved the decreased plasma estradiol concentration in ovariectomized rats and improved headache and depressive symptoms in middle-aged women (53, 54). The microvascular disorders index (MDI) was found to be related to menstrual and abdominal pain and associated with the menstrual cycle. Keishi-bukuryo-Gan (KBG) is used for menstrual pain *via* improving the inflammatory factors and anti-platelet aggregation (27). The meteoropathy-related index (MRI) means the distribution and circulation of body fluid. In fact, Gorei-San prevents brain edema by inhibiting the upregulation of aquaporin 4 in mice (55).

Previously, we developed a new simple frailty screening scale (Japan Frailty Scale: JFS) based on the aging concept of Kampo medicine [(56) submitted data]. The JFS showed high sensitivity as a screening tool for pre-frailty or frailty in elderly persons. At the same time, the JFS was well correlated with depression in elderly persons compared to the Geriatric Depression Scale (GDS). Thus, physical evaluation based on Kampo medicine might be a screening tool for psychological states.

Various factors have been discussed as the cause of postpartum depression. One of the biological factors is dramatic changes in hormone levels associated with breastfeeding and resumption of menstruation (57). From the psychological aspects, history of depression, prenatal depression and anxiety, and self-esteem are said to be related to postpartum depression (58). From the social aspects, dramatic changes in daily life due to child-rearing and household chores have been mentioned (59). Furthermore, life stress with childcare, childcare support system, marital status, and relationships are related to postpartum depression (60). In other words, postpartum depression is considered to develop from multidimensional aspects, the same as frailty in elderly people (61). Therefore, it is noteworthy that the present study showed the effectiveness of screening for depression based on Kampo medicine, which allows for a simple and multidimensional evaluation of the physical aspects.

The Kampo diagnostic concept of “sho” is often used for the prescription decision of the Kampo formula for an individual patient. Each clinical question contributes slightly to the prescription decision of the Kampo formula. However, problematic points are clustered and produce the diagnosis from the Kampo medicine point of view. In the present study, on multivariable logistic regression analysis, of the 17 questions in the MDPS, only two questions showed a significant relationship with the BDI-II, but when summarized in five subcategories, three categories (PAI, SDI, and HAI) showed significant relationships with the BDI-II, and the total score of the MDPS showed a significant relationship with the BDI-II. Recently, it has been reported that genetic polymorphisms contribute to the onset of frailty (62, 63). Similarly, polygenic risk scores are expected to predict risk assessment in clinical psychiatry when combined with data from molecular, clinical, and lifestyle metrics (64). In addition, gut microbiota have been reported to be related to depression and anxiety disorders and short-chain fatty acids that are important for maintaining the barrier function of the intestinal epithelium (65). It is thought that the inflammatory state affects the signal transmission of neurotransmitters, such as dopamine, norepinephrine, and serotonin, reward prediction, and action selection by the basal ganglia. As a result, decreased motivation is caused by worsening island function in the brain due to inflammation (66). MDPS-M might help make a more accurate diagnosis when combined with the polygenic score, and assessments of the gut microbiota and inflammatory cytokines.

This study has some limitations. First, this study used the BDI-II as the standard for depression. In general, the EPDS is used to test for postpartum depression. Credibility of the EPDS depends on privacy, a relaxed environment for women, and the childcare environment (23). In the present study, the BDI-II was appropriate for examination of mothers in various childcare situations. However, comparative studies with other depression scales are needed to better validate the accuracy of the MDPS-M in the future. Second, the relationship between maternal depression and physical symptoms was investigated using the MDPS-M. In the future, sufficient data of men with depression are needed to generalize whether depression can be predicted using the MDPS.

In conclusion, the MDPS-M was developed as a simple, self-reported, screening scale for mild depression from physical aspects, based on the theory of “Qi, Blood, and Fluid” in Kampo medicine. It is composed of 17 questions and resumption of menstruation. The combination of these symptoms is novel, and it is possibly an important phenotype of mothers with mild depression in the parenting period. The MDPS-M is a useful screening test for mild depression associated with childcare in Japanese child-rearing women.

## Data availability statement

The datasets generated and analyzed during the current study are available from the corresponding author on reasonable request.

## Ethics statement

The studies involving human participants were reviewed and approved by the Institutional Review Board of Osaka University School of Medicine (20521) and the Kyoto University Graduate School and Faculty of Medicine, Ethics Committee (R2624). The patients/participants provided their written informed consent to participate in this study.

## Author contributions

MT: writing – original draft, methodology, visualization, data curation, and investigation. MMa: writing – original draft, methodology, software, data curation, and investigation. RE: writing – original draft and visualization. AM: methodology, software, validation, and formal analysis. FY: writing – review. MN, MMo, ST, SH, HH, and HS: investigation. MMy: conceptualization, methodology, supervision, project administration, and funding acquisition. KH: conceptualization, methodology, writing – review and editing, visualization, supervision, project administration, and funding acquisition. All authors contributed to the article and approved the submitted version.

## Abbreviations

AIC: akaike information criterion
AUC: area under the curve
BDI-II: beck depression inventory–second edition
CI: confidence interval
EPDS: edinburgh postnatal depression scale
HAI: hormone activity index
HKT: hange-koboku-To
JFS: Japan frailty scale
KBG: keishi-bukuryo-Gan
MDI: microvascular disorder index
MDPS: multidimensional physical scale
MDPS-M: MDPS for mothers
MRI: meteoropathy related index
NPV: negative predictive value
OR: odds ratio
PAI: physical activity index
PPV: positive predictive value
RKT: rikkunshi-To
ROC: receiver-operating characteristic
SD: standard deviation
SDI: somatic disorders index
TSS: toki-shakuyaku-San.
